# Supplementary material for: Engineering a tunable bicistronic TetR autoregulation expression system in Gluconobacter oxydans
Source: PeerJ. 2022 Jul 19;10:e13639. doi: 10.7717/peerj.13639 (PMC9306550; doi:10.7717/peerj.13639)
Supplement: Supplemental Information 1 [file peerj-10-13639-s001.docx]

#Example code used to analyze all data sets and plot promoter activities.

library(tidyverse)

library(readxl)

library(glue)

library(ggtext)

library(patchwork)

luxp0169 <- read_excel("LUXp0169.xlsx")

luxp264 <- read_excel("LUXp264.xlsx")

luxp452 <- read_excel("LUXp452.xlsx")

#p0169 summary stats

mean(subset(luxp0169, Treatment == "p0169PLUX.UidA.0.0.uM")$Activity)

sd(subset(luxp0169, Treatment == "p0169PLUX.UidA.0.0.uM")$Activity)

mean(subset(luxp0169, Treatment == "p0169PLUX.UidA.0.1.uM")$Activity)

sd(subset(luxp0169, Treatment == "p0169PLUX.UidA.0.1.uM")$Activity)

mean(subset(luxp0169, Treatment == "p0169PLUX.UidA.0.25.uM")$Activity)

sd(subset(luxp0169, Treatment == "p0169PLUX.UidA.0.25.uM")$Activity)

mean(subset(luxp0169, Treatment == "p0169PLUX.UidA.0.5.uM")$Activity)

sd(subset(luxp0169, Treatment == "p0169PLUX.UidA.0.5.uM")$Activity)

mean(subset(luxp0169, Treatment == "p0169PLUX.UidA.1.uM")$Activity)

sd(subset(luxp0169, Treatment == "p0169PLUX.UidA.1.uM")$Activity)

mean(subset(luxp0169, Treatment == "p0169PLUX.UidA.5.uM")$Activity)

sd(subset(luxp0169, Treatment == "p0169PLUX.UidA.5.uM")$Activity)

mean(subset(luxp0169, Treatment == "p0169PLUX.UidA.ten.uM")$Activity)

sd(subset(luxp0169, Treatment == "p0169PLUX.UidA.ten.uM")$Activity)

#p264 summary stats

mean(subset(luxp264, Treatment == "p264PLUX.UidA.0.0.uM")$Activity)

sd(subset(luxp264, Treatment == "p264PLUX.UidA.0.0.uM")$Activity)

mean(subset(luxp264, Treatment == "p264PLUX.UidA.0.1.uM")$Activity)

sd(subset(luxp264, Treatment == "p264PLUX.UidA.0.1.uM")$Activity)

mean(subset(luxp264, Treatment == "p264PLUX.UidA.0.25.uM")$Activity)

sd(subset(luxp264, Treatment == "p264PLUX.UidA.0.25.uM")$Activity)

mean(subset(luxp264, Treatment == "p264PLUX.UidA.0.5.uM")$Activity)

sd(subset(luxp264, Treatment == "p264PLUX.UidA.0.5.uM")$Activity)

mean(subset(luxp264, Treatment == "p264PLUX.UidA.1.uM")$Activity)

sd(subset(luxp264, Treatment == "p264PLUX.UidA.1.uM")$Activity)

mean(subset(luxp264, Treatment == "p264PLUX.UidA.5.uM")$Activity)

sd(subset(luxp264, Treatment == "p264PLUX.UidA.5.uM")$Activity)

mean(subset(luxp264, Treatment == "p264PLUX.UidA.ten.uM")$Activity)

sd(subset(luxp264, Treatment == "p264PLUX.UidA.ten.uM")$Activity)

#p452 summary stats

mean(subset(luxp452, Treatment == "p452PLUX.UidA.0.0.uM")$Activity)

sd(subset(luxp452, Treatment == "p452PLUX.UidA.0.0.uM")$Activity)

mean(subset(luxp452, Treatment == "p452PLUX.UidA.0.1.uM")$Activity)

sd(subset(luxp452, Treatment == "p452PLUX.UidA.0.1.uM")$Activity)

mean(subset(luxp452, Treatment == "p452PLUX.UidA.0.25.uM")$Activity)

sd(subset(luxp452, Treatment == "p452PLUX.UidA.0.25.uM")$Activity)

mean(subset(luxp452, Treatment == "p452PLUX.UidA.0.5.uM")$Activity)

sd(subset(luxp452, Treatment == "p452PLUX.UidA.0.5.uM")$Activity)

mean(subset(luxp452, Treatment == "p452PLUX.UidA.1.uM")$Activity)

sd(subset(luxp452, Treatment == "p452PLUX.UidA.1.uM")$Activity)

mean(subset(luxp452, Treatment == "p452PLUX.UidA.5.uM")$Activity)

sd(subset(luxp452, Treatment == "p452PLUX.UidA.5.uM")$Activity)

mean(subset(luxp452, Treatment == "p452PLUX.UidA.ten.uM")$Activity)

sd(subset(luxp452, Treatment == "p452PLUX.UidA.ten.uM")$Activity)

cbPalette <- c("#999999", "#E69F00", "#56B4E9", "#009E73", "#F0E442", "#0072B2", "#D55E00", "#CC79A7")

#non-parmetric test

ktp0169 <- kruskal.test(Activity ~ Treatment, data=luxp0169)

ktp0169$p.value

ktp264 <- kruskal.test(Activity ~ Treatment, data=luxp264)

ktp264$p.value

ktp452 <- kruskal.test(Activity ~ Treatment, data=luxp452)

ktp452$p.value

#if p < 0.5 then you can do the pairwise and add bars. Make if to automate if satisfies condition

if(ktp0169$p.value < 0.5){

pt_p0169 <- pairwise.wilcox.test(luxp0169$Activity,

g=luxp0169$Treatment,

p.adjust.method = "BH")}

pt_p0169$p.value

if(ktp264$p.value < 0.5){

pt_p264 <- pairwise.wilcox.test(luxp264$Activity,

g=luxp264$Treatment,

p.adjust.method = "BH")}

pt_p264$p.value

if(ktp452$p.value < 0.5){

pt_p452 <- pairwise.wilcox.test(luxp452$Activity,

g=luxp452$Treatment,

p.adjust.method = "BH")}

pt_p452$p.value

#Make figures

box_plot_p0169 <- luxp0169 %>%

ggplot(aes(x=Treatment, y=Activity, fill=Treatment)) +

stat_summary(fun = median, show.legend=FALSE, geom="crossbar") +

geom_boxplot(outlier.color = "white", outlier.shape = 16, outlier.size = 2, fill = "white", notch = FALSE) +

geom_jitter(show.legend=FALSE, width=0.25, shape=21, color="black") +

labs(x=NULL,

y="Miller Units") +

scale_x_discrete(breaks=c('p0169PLUX.UidA.0.0.uM','p0169PLUX.UidA.0.1.uM', 'p0169PLUX.UidA.0.25.uM', 'p0169PLUX.UidA.0.5.uM','p0169PLUX.UidA.1.uM', 'p0169PLUX.UidA.5.uM', 'p0169PLUX.UidA.ten.uM'),

labels=c(glue("p0169<br>0"),

glue("p0169<br>0.1"),

glue("p0169<br>0.25"),

glue("p0169<br>0.5"),

glue("p0169<br>1"),

glue("p0169<br>5"),

glue("p0169<br>10"))

) +

scale_fill_manual(name=NULL,

breaks=c('p0169PLUX.UidA.0.0.uM','p0169PLUX.UidA.0.1.uM', 'p0169PLUX.UidA.0.25.uM', 'p0169PLUX.UidA.0.5.uM','p0169PLUX.UidA.1.uM', 'p0169PLUX.UidA.5.uM', 'p0169PLUX.UidA.ten.uM'),

values= cbPalette) +

xlab(expression(paste("AHL (",mu,"M)"))) +

ylim(0,12000) +

theme_classic() +

theme(axis.text.x = element_markdown())

box_plot_p0169

p0169_plot <- box_plot_p0169 +

geom_line(data=tibble(x=c(1,2), y=c(11570,11570)),

aes(x=x, y=y),

inherit.aes=FALSE) +

geom_text(data=tibble(x=1.5, y=11570),

aes(x=x, y=y, label = '*'),size = 8,

inherit.aes=FALSE)

p0169_plot

box_plot_p264 <- luxp264 %>%

ggplot(aes(x=Treatment, y=Activity, fill=Treatment)) +

stat_summary(fun = median, show.legend=FALSE, geom="crossbar") +

geom_boxplot(outlier.color = "white", outlier.shape = 16, outlier.size = 2, fill = "white", notch = FALSE) +

geom_jitter(show.legend=FALSE, width=0.25, shape=21, color="black") +

labs(x=NULL,

y="Miller Units") +

scale_x_discrete(breaks=c('p264PLUX.UidA.0.0.uM','p264PLUX.UidA.0.1.uM', 'p264PLUX.UidA.0.25.uM', 'p264PLUX.UidA.0.5.uM','p264PLUX.UidA.1.uM', 'p264PLUX.UidA.5.uM', 'p264PLUX.UidA.ten.uM'),

labels=c(glue("p264<br>0"),

glue("p264<br>0.1"),

glue("p264<br>0.25"),

glue("p264<br>0.5"),

glue("p264<br>1"),

glue("p264<br>5"),

glue("p264<br>10"))

) +

scale_fill_manual(name=NULL,

breaks=c('p264PLUX.UidA.0.0.uM','p264PLUX.UidA.0.1.uM', 'p264PLUX.UidA.0.25.uM', 'p264PLUX.UidA.0.5.uM','p264PLUX.UidA.1.uM', 'p264PLUX.UidA.5.uM', 'p264PLUX.UidA.ten.uM'),

values= cbPalette) +

xlab(expression(paste("AHL (",mu,"M)"))) +

ylim(0,12000) +

theme_classic() +

theme(axis.text.x = element_markdown())

box_plot_p264

p264_plot <- box_plot_p264 +

geom_line(data=tibble(x=c(1,2), y=c(5650,5650)),

aes(x=x, y=y),

inherit.aes=FALSE) +

geom_text(data=tibble(x=1.5, y=5650),

aes(x=x, y=y, label = '*'),size = 8,

inherit.aes=FALSE)

p264_plot

box_plot_p452 <- luxp452 %>%

ggplot(aes(x=Treatment, y=Activity, fill=Treatment)) +

stat_summary(fun = median, show.legend=FALSE, geom="crossbar") +

geom_boxplot(outlier.color = "white", outlier.shape = 16, outlier.size = 2, fill = "white", notch = FALSE) +

geom_jitter(show.legend=FALSE, width=0.25, shape=21, color="black") +

labs(x=NULL,

y="Miller Units") +

scale_x_discrete(breaks=c('p452PLUX.UidA.0.0.uM','p452PLUX.UidA.0.1.uM', 'p452PLUX.UidA.0.25.uM', 'p452PLUX.UidA.0.5.uM','p452PLUX.UidA.1.uM', 'p452PLUX.UidA.5.uM', 'p452PLUX.UidA.ten.uM'),

labels=c(glue("p452<br>0"),

glue("p452<br>0.1"),

glue("p452<br>0.25"),

glue("p452<br>0.5"),

glue("p452<br>1"),

glue("p452<br>5"),

glue("p452<br>10"))

) +

scale_fill_manual(name=NULL,

breaks=c('p452PLUX.UidA.0.0.uM','p452PLUX.UidA.0.1.uM', 'p452PLUX.UidA.0.25.uM', 'p452PLUX.UidA.0.5.uM','p452PLUX.UidA.1.uM', 'p452PLUX.UidA.5.uM', 'p452PLUX.UidA.ten.uM'),

values= cbPalette) +

xlab(expression(paste("AHL (",mu,"M)"))) +

ylim(0,12000) +

theme_classic() +

theme(axis.text.x = element_markdown())

box_plot_p452

p452_plot <- box_plot_p452 +

geom_line(data=tibble(x=c(1,2), y=c(5550,5550)),

aes(x=x, y=y),

inherit.aes=FALSE) +

geom_text(data=tibble(x=1.5, y=5600),

aes(x=x, y=y, label = '*'),size = 8,

inherit.aes=FALSE)

p452_plot

(p0169_plot + p264_plot + p452_plot) + plot_annotation(title = "", tag_levels = "A") &

theme(plot.tag = element_text(face="bold"))

ggsave('LUX_Combined_Fig.png', width = 12, height = 3.5)
